# Trunk Surgery as a Tool to Reduce Foliar Symptoms in Diseases of the Esca Complex and Its Influence on Vine Wood Microbiota

**DOI:** 10.3390/jof7070521

**Published:** 2021-06-29

**Authors:** Andrea Pacetti, Samuele Moretti, Catia Pinto, Stéphane Compant, Sibylle Farine, Christophe Bertsch, Laura Mugnai

**Affiliations:** 1 Department of Agricultural, Food, Environmental and Forestry Science and Technology (DAGRI), Plant Pathology and Entomology Section, University of Florence, P.le delle Cascine 28, 50144 Firenze, Italy; samuele.moretti@uha.fr (S.M.); laura.mugnai@unifi.it (L.M.); 2 Laboratoire Vigne Biotechnologies et Environnement (LVBE, UR-3991), Université de Haute Alsace, 33 Rue de Herrlisheim, CEDEX, 68000 Colmar, France; sibylle.farine@uha.fr (S.F.); christophe.bertsch@uha.fr (C.B.); 3 Biome Makers, 890 Embarcadero Drive, West Sacramento, CA 95605, USA; catia_carvalho_pinto@yahoo.com; 4 AIT Austrian Institute of Technology GmbH, Center for Health & Bioresources, Bioresources Unit, Konrad Lorenz Straße 24, 3430 Tulln, Austria; stephane.compant@ait.ac.at

**Keywords:** curettage, *Fomitiporia mediterranea*, *Phaeomoniella chlamydospora*, grapevine, decay

## Abstract

In the last few years, trunk surgery has gained increasing attention as a method to reduce foliar symptoms typical of some of the Esca complex diseases. The technique relies on the mechanical removal of decayed wood by a chainsaw. A study on a 14-year-old Cabernet Sauvignon vineyard was carried out to validate the efficacy of trunk surgery and explore possible explanations behind it. Three levels of treatment were applied to three of the most characteristic symptoms associated with some diseases of the Esca complex, such as leaf stripe symptoms (LS), wilted shoots (WS) and apoplexy (APP). The most promising results were obtained by complete trunk surgery, where the larger decay removal allowed lower symptom re-expression. According to the wood types analyzed (decay, medium and sound wood), different changes in microbiota were observed. Alpha-diversity generally decreased for bacteria and increased for fungi. More specifically, main changes were observed for *Fomitiporia mediterranea* abundance that decreased considerably after trunk surgery. A possible explanation for LS symptom reduction after trunk surgery could be the microbiota shifting caused by the technique itself affecting a microbic-shared biochemical pathway involved in symptom expression.

## 1. Introduction

Wood diseases are a major threat for modern viticulture, and the diseases included within the Esca complex remain the most worrying in Europe and worldwide [1,2]. Esca complex is currently considered a complex of different diseases and syndromes (characterized by several different symptoms) mostly associated with ascomycetes species, namely vascular pathogens as *Phaeomoniella chlamydospora* (W. Gams, Crous, M.J. Wingf. & Mugnai) [3] (Pch), *Phaeoacremonium minimum* (syn. *P. aleophilum*) (Tul. & C. Tul.) [4] (Pmin), and basidiomycetes species, which in Europe are mostly represented by the white rot agent [5] *Fomitiporia mediterranea* [6] (Fmed). The role of Botryosphaeriaceous species in some of the Esca complex diseases is also frequently investigated [7,8]. Following the definition proposed by Surico [9], the Esca complex includes four diseases: brown wood streaking, grapevine leaf stripe disease (GLSD), Petri disease—all associated with vascular pathogens—and Esca, the wood white rot that originally was named as such. When both GLSD foliar symptoms and white rot are present, the term “Esca proper” can be used to describe this condition [9,10,11,12]. *Fomitiporia mediterranea* (originally misidentified as *F. punctata*) [13], was shown to be capable of colonizing the wood as a primary pathogen [14,15]. Despite this ability, the assumption that Fmed colonization of wood can be facilitated by a pre-colonization of other pioneer pathogens has not yet been disproved [10,16,17]. The external symptoms detectable in vineyards are the leaf stripe symptoms described by many authors [10,18,19,20], the apoplectic stroke [21], which originally was considered as synonymous with “Esca disease” [22,23] and the shoots and clusters wilting, which in literature is attributed mainly to Esca complex diseases [11,21,24]. The external symptoms develop more and more frequently as the vines get older and multiple wood pathogens can be associated with the foliar symptoms [25,26,27,28]. The association of the same foliar symptoms with such a variable mycoflora and the failure of the Koch’s postulates partially explains why the factors triggering the leaf stripe symptoms and wilting have not been fully clarified despite the many hypotheses formulated [29]. During the last two decades, different approaches to manage leaf stripe symptoms and to reduce vine death due to diseases of Esca complex have been tested and applied in the field. After the phasing out of sodium arsenite in the early 2000s, the applied tools for managing leaf stripe symptoms have been diversified, including pruning timing and modes, wound protection, remedial surgery and foliar treatments using biostimulants or chemical products [30,31,32,33]. Among the remedial surgery treatments, trunk renewal and trunk surgery are the best-known methods. Trunk renewal was primarily used against Eutypa dieback and Botryosphaeria dieback and is defined as an inexpensive and relatively easy approach to apply [34,35]. Trunk surgery is a long-used approach on fruit trees in the Mediterranean area and has been recently re-discovered. It is also named “slupatura” or “curetage du bois”, in Italy or France, respectively. The aim of this technique is to quickly recover productivity of symptomatic plants by keeping their active root systems and therefore maintaining the quality of the product that is linked to the vine age. The technique is carried out using an electric or gas chainsaw with a mounted carving blade. First, to identify the rotten areas, a preliminary cut is done either where dead wood emerges or is found under the biggest pruning wounds. The plant trunk is opened to be inspected. The decay, often located in the upper part of the vine where the larger wounds are applied, is then removed. The actual applicability of the technique is very much linked to the pruning system (Guyot being the ideal pruning system for trunk surgery). Decay removal consists of scraping out the fibrous and bleached decayed wood, leaving the discolored brown wood and the sound wood. It is reported that the removal of brown wood does not affect the result of the technique, and that it is important not to compromise the main sap flow during the trunk surgery, preferring to act only on one side of the plant [36,37].

Trunk surgery is considered more expensive and time-consuming compared to trunk renewal [31] and needs well-trained personnel. Following the description reported in the available literature, the total removal of decayed wood has a great influence on leaf stripe symptom suppression. Moreover, it seems that the decay found in the trunk has less influence on leaf stripe symptom expression than the decay located in the upper part of the vine trunk [36,37]. Despite the promising results brought to light, currently there is a lack of data in the scientific literature about this technique, and only results of some field trials are available in technical magazines and conference proceedings [37,38,39]. No description of the changes induced in the plant by the rotten tissue removal operation are reported. No explanation has yet been formulated on the changes in the plant–pathogen interaction that bring trunk surgery to reduce foliar symptoms. The exposure of the active wood to air and light could cause a change in the remaining sound wood-microflora composition or activity. Alternatively, the removal of the decayed tissue could reduce the amount of wood degrading enzymes. Approaches for studying changes in the microflora have been recently utilized more frequently, with metabarcoding as one of the most promising methodologies to screen the entire microbiota [26,40,41]. In this study, we investigate the efficacy of trunk surgery in reducing the external symptoms linked to some diseases of the Esca complex and on the possible role of decayed wood removal on the microbiota in the trunk of Esca complex affected vines. The ultimate goal is to implement the knowledge on the factors involved in the expression of foliar symptoms, for which some hypotheses have already been formulated [29] but without being able to replicate the complete symptomatologic picture.

## 2. Materials and Methods

### 2.1. The Vineyard

This study was carried out in a 14-year-old vineyard located in Tuscany, one of the most important winegrowing areas in Italy (42°57′16.9″ N, 11°02′44.3″ E). According to Köppen–Geiger climate classification [42], this area is characterized by a warm temperate climate, with a hot and dry summer [43]. The vineyard was planted in 2004 using *Vitis vinifera* L., cv. “Cabernet Sauvignon”, clone R5 omega-grafted onto 161.49 rootstock (*Vitis berlandieri* × *Vitis riparia*) with a density of 4,350 plants/ha at plantation time. The vineyard is located at an altitude of 50 m, exposition west with rows W-E oriented. The training system was a spur pruned cordon for 9 years then it was converted to single Guyot Poussard. This conversion caused several wide-diameter cuts and typical leaf stripe symptoms and apoplectic vines reached 37% of the coetaneous vines standing in the year before this study. An integrated pest management program was adopted to control foliar diseases (e.g., downy mildew, powdery mildew, gray mold) but no chemical or biological treatment against wood diseases was ever applied in the vineyard during the period of study.

### 2.2. Treatments

**Figure 1:**
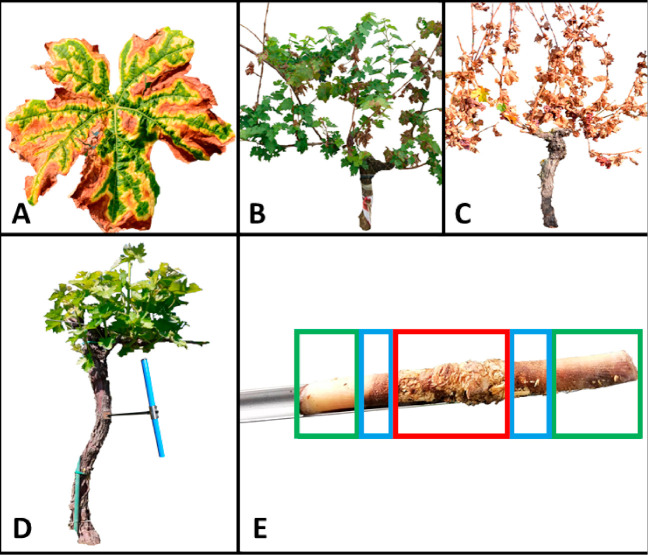
Symptoms monitored during the survey and sampling mode. (**A**) Leaf stripe symptoms (LS) involve leaves that show a green band around the main veins with a chlorotic perimeter while the central interveinal area appears reddish or yellow depending on the variety; in most cases this latter area progressively necrotizes. The necrosis often reaches the leaf margin. The necrotic foliar tissue appears with different colors depending on the variety, from red-brick to dark purple or brown. (**B**) Wilted shoots symptoms (WS) involve entire canes which wilt and necrotize entirely. Leaves wilt and dry up remaining attached to the cane. One or many shoots can dry out and wilt on the same plant, but the vine remains alive. Wilted canes appear shriveled and dry. This symptom is shown mostly during late summer and, if clusters are present, they also dry up remaining attached to the cane. (**C**) Apoplexy (APO) symptom consists in a complete wilt of the vine during summer. All canes, leaves and clusters dry up remaining attached to the canes and the plant apparently dies. During the late part of August or in September or the year after, some of the apoplectic vines resumed some partial and weak growth. (**D**) Sampling procedure using an increment borer, performed on the central portion of the trunk. (**E**) The three types of wood sampled: sound wood (green box), median wood (blue box) and decayed wood (red box).

To evaluate the efficacy of trunk surgery, in July 2018 the vineyard was mapped, and each vine was classified on the base of the symptoms recorded during the survey. Three symptom types were selected: (i) leaf stripe symptoms (LS), (ii) wilted shoots (WS) and (iii) apoplexy (APP) as shown in Figure 1A–C, respectively. Coding was used to evaluate symptoms before and after trunk surgery. Treatments were performed right after the first survey at the end of July 2018 (summer-treatments) and in February 2019 (winter-treatments). Three level of treatments were tested to prove the effectiveness of the technique and to evaluate the relevance of the complete decay removal: classical trunk surgery (S), with a total removal of white decayed wood; a less invasive version here called half surgery (HS), where only the core of decayed wood tissue in trunk and in branch was removed; a side-by-side trunk trespassing cut (Cut) made with a chainsaw. Treatments were performed only on plants that had shown symptoms during the previous survey. Summer-treatments were performed on 90 LS-symptomatic vines (*n* = 30 per treatment level). For winter-treatments, all three symptom types were treated with three levels of trunk surgery each: 270 vines were treated (*n* = 30 per treatment level), while 90 more vines were monitored as untreated control (UTC) per each symptom type (*n* = 30 per symptom type). 

### 2.3. Wood Sampling for Microbiota Analysis

To analyze the changes induced by the treatment on the wood-associated microbiota, 3 vines treated by complete trunk surgery (S) were sampled. A specific sampling was performed on vines that had shown foliar LS symptoms in the previous season and were not involved in the on-field trial. Three vines were sampled immediately before treatment in May 2019 (T0), namely at flowering stage (BBCH 60), and 90 days after treatment (T3) (BBCH 83) when foliar symptoms are usually mostly expressed in field, only on the remaining median and sound wood. As control, three more vines were sampled at T0 and T3 without applying trunk surgery. Vines were sampled using an increment borer (which has already been suggested as a suitable tool to study GTDs by Muruamendiaraz and Legorburu [44], at each time point (Figure 1D). Each biological sample was made of three wood cores (subsamples) of 6 mm in diameter per each plant, at each time point. Samples were collected from the central part of the trunk (almost 30 cm above the soil); tools were carefully disinfected in between each sampling with 70% ethanol, to avoid microbiota cross-contamination. In each wood core, three wood types were identified and separated right after sampling: (i) decayed wood, (ii) median wood (the intermediate discolored, dark brown wood which normally forms between decayed and sound wood) and (iii) sound wood which is the apparently healthy wood (Figure 1E). Once separated by wood type, subsamples were bulked forming the samples to be analyzed and were put in an Eppendorf tube shielded from light and stored at −20°C until processing for microbiota analysis. For microbiota analysis, a total of 30 samples were analyzed for both bacterial and fungal communities, through 16S rRNA gene and ITS gene count data, respectively. 

### 2.4. Microbiota Analysis

#### 2.4.1. Sample Processing

Three biological samples for each type of wood, for treated and untreated conditions and at each time point, were analyzed. For each sample, a total of 25 mg of pulverized wood was used for the DNA extraction using the Danagene Microbiome Soil DNA kit (Danagene, Badalona, Spain), according to the manufacturer’s instructions. The yield and purity of the DNA was measured by Qubit and then stored at −20 °C until use. 

#### 2.4.2. Library Preparation and Sequencing

PCR reactions were prepared using UV sterilized equipment and negative controls containing sterile water were run alongside the samples. Samples were analyzed for the 16S rRNA gene V4 region, and the ITS gene by amplification of the ITS1 region using WineSeq^®^ custom primers accordingly to the Patent WO2017096385 [45]. After a quality control by gel electrophoresis, each library (16S rRNA gene and ITS genes) was pooled in equimolar amount and subsequently sequenced on an Illumina MiSeq instrument (Illumina, San Diego, CA, USA) using 2 × 301 paired-end reads and according to the Biome Makers implemented protocol. All the data produced and collected were subsequently analyzed through a QIIME-based custom and inhouse bioinformatics pipeline (Patent WO2017096385). A first quality control was used to remove adapters and chimeras [46] and after that, the reads were trimmed out from the point where these did not reach the appropriate quality score. Operational taxonomic unit (OTU) clusters were performed using 97% identity and taxonomy assignment and abundance estimation were obtained comparing OTUs clusters obtained with SILVA database, version 132 [47] and UNITE database version 7.2 [48] as taxonomic references. Results obtained were used to identify fungal and bacteria at species and/or genus taxonomic level.

#### 2.4.3. Statistical Analysis

Results of on-field trials were analyzed by performing contingency table analysis (Pearson chi-square test) and the residual z-scores were studied to establish statistical differences between groups (α = 0.05). A 4 × 4 (treatments × surveyed-symptom) contingency table was analyzed per each 2018-recorded symptom type. *p*-values were compared with Bonferroni’s adjusted *p*-value after its calculation based on standardized residual [49].

For wood microbiota analysis, the relative abundances of each sample were calculated for all taxa based on the total population identified. Taxa with less than 10 non-zero values were grouped into a single column labelled “Others”. The zero counts were replaced for all samples using a Bayesian method (with a Dirichlet multinomial prior), and a multiplicative replacement was performed to maintain the original ratios between the parts of the composition. Sample-wise differences were calculated using the Aitchison distance, and ordination was performed via Kruskal’s non-metric multidimensional scaling. Alpha and beta-diversity were analyzed separately for bacteria and fungi using OTUs counts. Alpha diversity was analyzed through Shannon’s index [50] and observed richness, which was calculated using three samples as biological repetitions and plotted against wood type groups; time and treatment factors were statistically analyzed using a two-way ANOVA test (α < 0.05) and Duncan post-hoc was performed where interaction between factor results were significant. Decayed wood was not considered in these analyses as decay was removed by the treatment applied. Regarding the beta-diversity, a non-metric multidimensional scaling (NMDS) analysis was performed to highlight clusters of samples for both fungal and bacterial microbiota. All analyses were performed in the R (3.6.3 version) programming environment.

## 3. Results

### 3.1. On-Field Results

Regarding the evaluation of the efficacy of trunk surgery on reducing the expression of the three selected Esca symptoms, the vineyard was surveyed in September 2019 and 2020. Total Esca incidence in the whole vineyard in 2019 was 14.3%. In 2020, a similar incidence rate was recorded (14.6%). 

#### 3.1.1. Summer-Treated Vines

**Figure 2:**
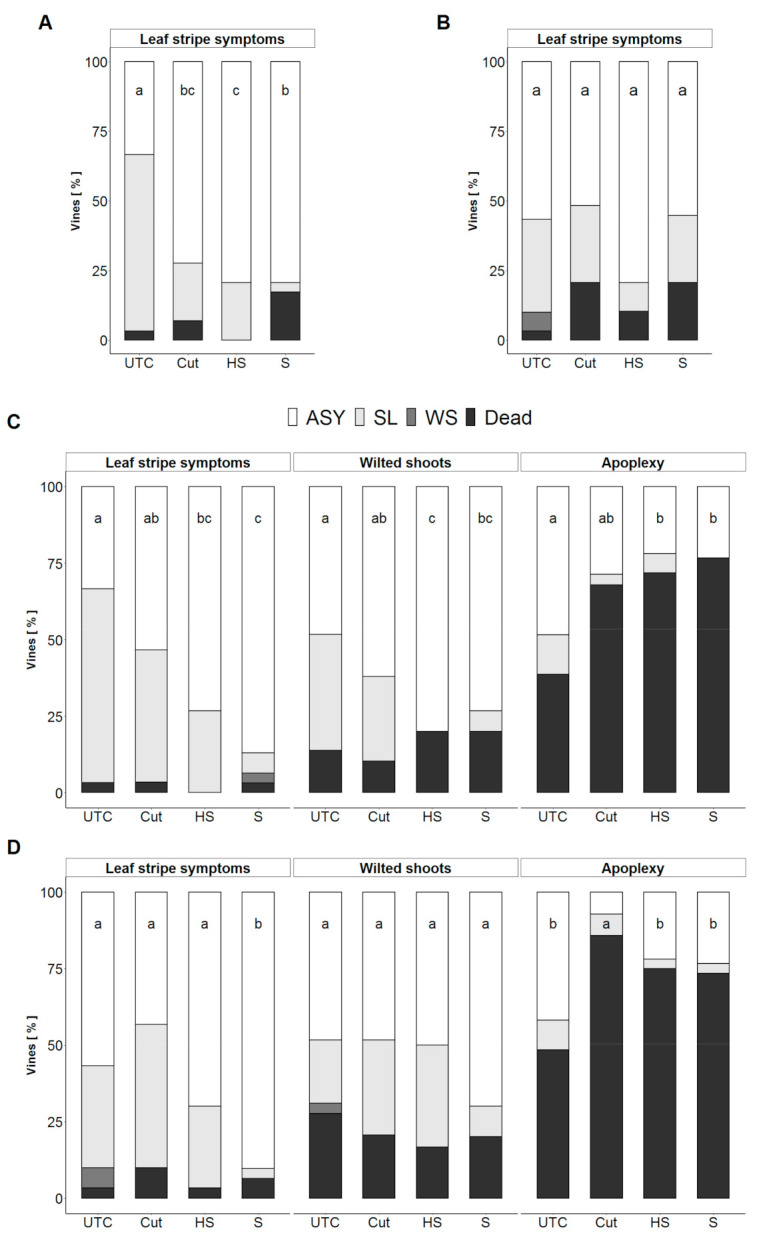
Health status of surveyed vines, one and two years after treatments. (**A**) Summer-treated vines surveyed one year after treatment (2019) and (**B**) two years after treatment (2020). (**C**) Winter-treated vines surveyed one year after treatments (2019) and (**D**) two years after treatment (2020). Histograms are grouped by the symptoms recorded before treatments (survey 2018). Black and grey shades represent symptoms reported in 2019 and 2020 for both untreated and treated plants: asymptomatic vines (ASY), striped leaf vines (LS), wilted cane vines (WS) and dead vines (Dead). The number of plants is reported as frequency. On X-axis, untreated control (UTC), trespassing cut (Cut), half surgery (HS) and complete trunk surgery (S) are the treatments reported and repeated per each 2018-monitored symptom type. Different letters on bars top represents statistically significant differences between treatments, considering all symptom types at once, according to Pearson chi-square test (α = 0.05); *p*-value were compared with Bonferroni’s adjusted *p*-value.

Summer-treatments were applied only on vines that showed LS symptoms during survey in July 2018. Obtained results are shown in Figure 2A,B. The LS re-expression of the untreated 2018-symptomatic vines was higher in 2019 (63%) than in 2020 (33%). Only one untreated vine died in each of the two surveyed years and two vines showed WS symptoms in 2020. All levels of treatment decreased the LS incidence compared to UTC in 2019 but these results were not confirmed in 2020. The re-expression of LS symptoms increased in Cut and S surgery levels two years after treatment 2020, while—despite LS symptoms did not increase in HS-treated vines—a 10% increase of dead vines was recorded from 2019 to 2020. Twenty-one percent of the total Cut-treated and S-treated vines died in the two years of survey.

#### 3.1.2. Winter-Treated Vines

Vines that showed LS-symptoms in 2018—considering the three treatments as increasing levels of decay removal, data highlight that symptoms occurred less frequently where the quantity of removed decay was greater. This was observed for both 2019 (Cut = 43%, HS = 27% and S = 6.5%) and 2020 (Cut = 47%, HS = 27% and S = 3%) (Figure 2C,D). Despite untreated vines showed less symptoms in 2020 compared to 2019, treated vine symptomatology kept stable over the two years confirming the results obtained. 

Vines that showed WS symptoms in 2018—WS symptoms were not re-expressed by untreated vines in 2019 and only one case was recorded in 2020 (Figure 2C,D). Over the two surveyed years, 48% of the untreated vines continued to grow asymptomatically. The rate of dead vines increased from 14% (2019) to 28% (2020). Fifteen percent (15%) of Cut-treated vines showed leaf stripe symptoms in 2019 and 20% in 2020. An increase of 10% of dead vines was recorded from 2019 to 2020 (Cut-treated vines). Vines treated with the complete surgery (S) showed a similar symptoms frame in both 2019 and 2020 (20% dead in both years, 6.7% SL-symptomatic in 2019 and 10% of SL-symptomatic in 2020) and 33% of vines treated with the half surgery showed LS symptoms in 2020 when no symptomatic vine among these vines was recorded in 2019. Twenty percent (20%) of HS-treated vines died in 2019 and one of these vines restarted growing again asymptomatically in 2020.

Vines that showed APO symptoms in 2018—sixty-one percent (61%) of apoplectic untreated vines restarted growing in 2019; 13% of those showed leaf stripe symptoms. A high percentage of untreated vines which showed apoplexy symptoms (APO) in 2018 died during the survey period (39% in 2019 and 48% in 2020) and almost the same percentage kept showing leaf stripe symptoms in both years. The dead vine number increased considerably in all treatments compared to untreated control in both 2019 (Cut = 68%, HS = 72% and S = 77%) (Figure 2C) and 2020 (Cut = 86%, HS = 75% and S = 73%) (Figure 2D).

### 3.2. Microbiota Overview

**Figure 3:**
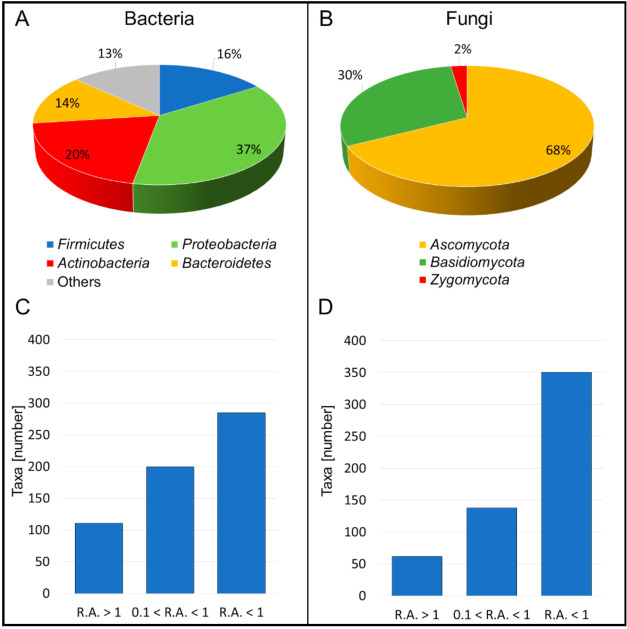
Number of bacterial taxa (**A**) and fungal taxa (**B**) identified referring to SILVA and UNITE taxonomy database according to Materials and Methods description. For both bacteria (**C**) and fungi (**D**), the composition in phyla and the distribution of identified taxa grouped by class of relative abundance (R.A.) are presented.

The deep sequencing of microbial communities originated a total of 3,180,890 high quality reads. For eukaryotic microorganisms 1,948,262 sequences were obtained and 1,232,628 for prokaryotes. All the high-quality sequence reads were grouped at a genetic distance of 3% and generated a total of 142 OTUs for ITS1, and 5140 for 16S. On average, 117 ± 37 and 921 ± 274 OTUs were obtained for eukaryotes and prokaryotes, respectively. Regarding the taxonomy assignment, a total of 553 fungal and 596 bacterial taxa (genus or species) were identified (Figure 3A,B). Sixty-two fungal taxa and 111 bacterial taxa were found in a relative abundance greater than 1% in at least one sample, while 138 fungal taxa and 200 bacterial taxa were detected with a relative abundance between 0.1% and 1% (Figure 3C,D). Taxa belonging to the phylum *Ascomycota* represent 68% of the eukaryotic microbiota, while 30% belong to *Basidiomycota* and 2% to *Zygomycota* (Figure 3B). The prokaryotic microbiota was represented by 21 phyla of which *Proteobacteria* (37%), *Actinobacteria* (20%), *Firmicutes* (16%) and *Bacteroidetes* (14%) were the most abundant (Figure 3A).

#### 3.2.1. Microbiota Composition of Wood Types

**Figure 4:**
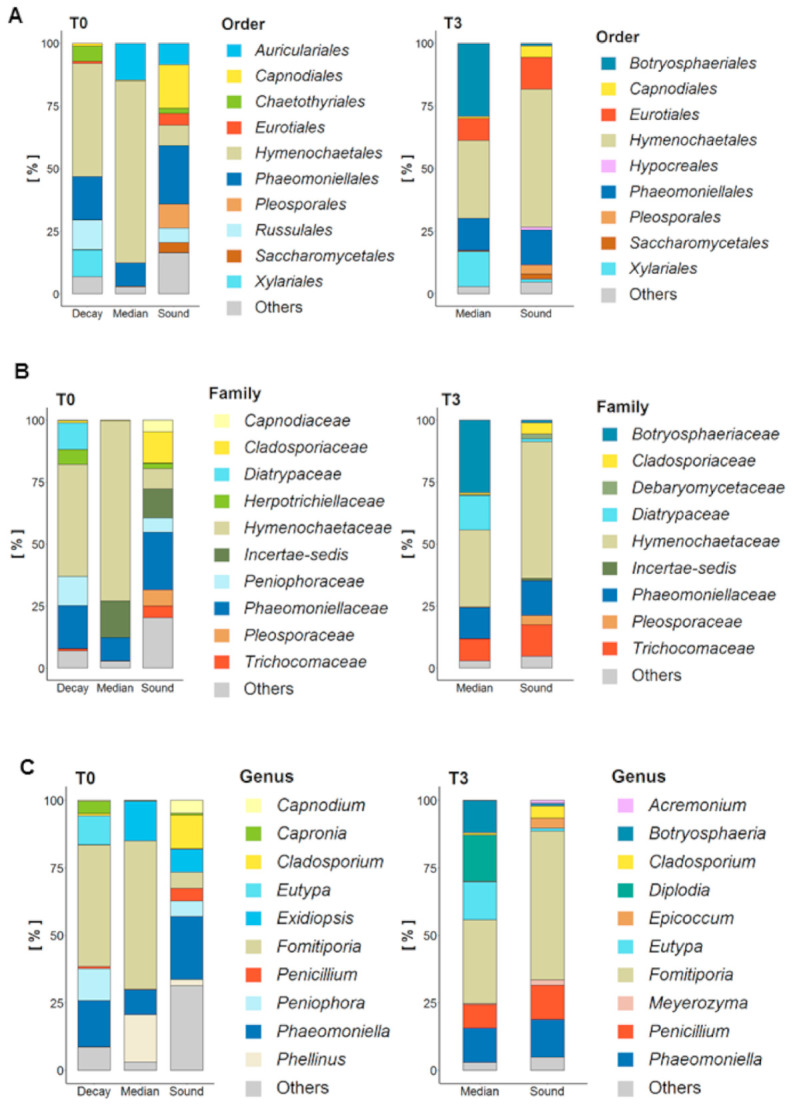
Overview of fungal microbiota composition of untreated vines (UTC) at T0 (May 2019) and T3 (July 2019). Decay, median and sound wood at T0 and median and sound wood at T3 were considered and order (**A**), family (**B**) and genus (**C**) compared. Samples were sampled from LS-diseased vines, which showed symptoms the year before (2018). The means of three biological repetitions per each wood type were calculated. Only taxa with relative abundance higher than 1% are showed.

Fungal microbiota composition was analyzed considering decay, median and sound wood at T0 and median and sound wood at T3 (Figure 4A–C; Appendix A, Table A1). Microbiota composition of decayed wood at T0, was mainly represented by *Basidiomycota*, namely the order *Hymenochaetales* (45%), which consisted almost completely of *Fomitiporia mediterranea*, and *Russulales* (12%), namely *Peniophora* genus (Figure 4A,C). The most abundant *Ascomycota* genera present in decayed wood were *Phaeomoniella* (17%) and *Eutypa* (11%), followed by *Capronia*, which belong to and completely represent the *Herpotrichiellaceae* family (6%) (Figure 4B,C). Among the genus *Phaeomoniella*, only Pch was identified and only *Eutypa lata* was found in the genus *Eutypa*. *Phellinus mori* was detected in relevant abundance (18%) in median wood at T0 where 54% of fungal diversity was represented by Fmed, 9% by Pch and 15% by *Auriculariales* order. At T3, in median wood *Penicillium* spp. and species in *Botryosphaeriaceae* were consistently detected while Fmed was the only *Basidiomycota* still present in significant amounts. Microbiota composition of sound wood was more diverse at T0 than at T3: *Phaeomoniellales* (here only Pch) represented the 23% of diversity at T0. Fmed increased considerably from T0 to T3 in sound wood (from 5% to 55%). As observed in median wood, also in sound wood *Botryosphaeriaceae* were detected only at T3 (Figure 4B). Sound wood at T0 showed the higher microbiota diversity compared to the other wood types and to sound wood itself at T3. Curiously, members of *Saccharomycetales* were found only in sound wood at both T0 and T3. Despite the fact that more than 500 fungal taxa were identified in grapevine trunk wood, more than 50% of the diversity was attributed to only a few species, and mostly associated with pathogenic microorganisms (Supporting Material Table A1).

**Figure 5:**
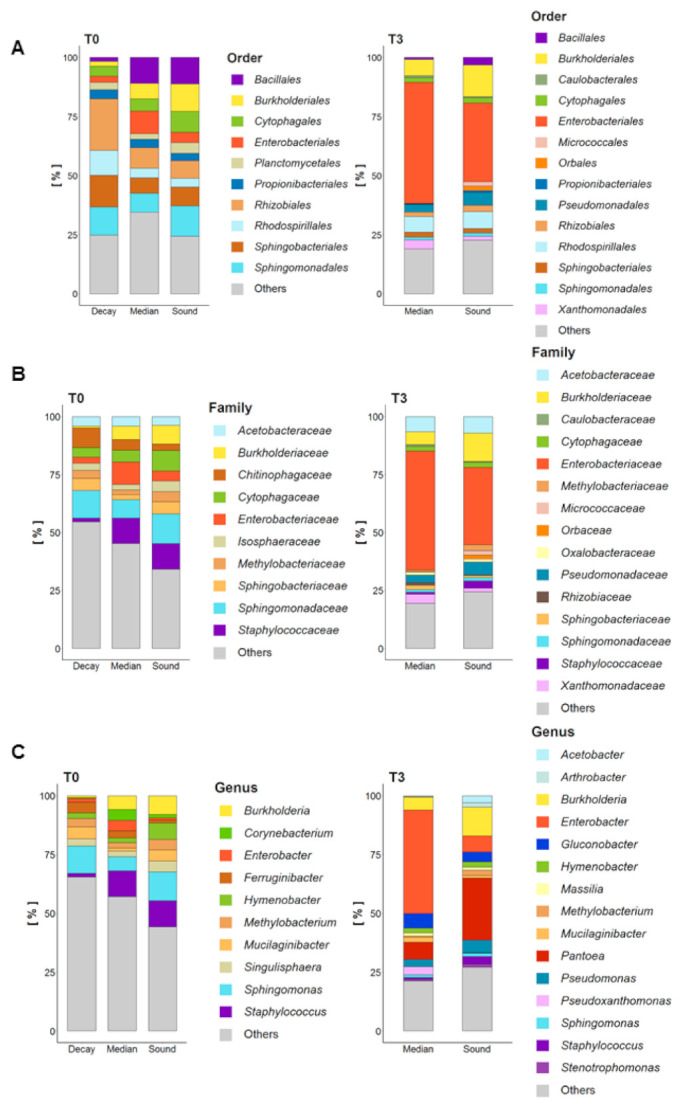
Overview of bacterial microbiota composition of untreated vines at T0 (May 2019) and T3 (July 2019). Decay, median and sound wood at T0 and median and sound wood at T3 were considered and order (**A**), family (**B**) and genus (**C**) compared. Samples were sampled from LS-diseased vines which showed symptoms in the previous year (2018). The mean of three biological repetitions per each wood type was calculated. Only taxa with relative abundance higher than 1% are showed.

Regarding the bacterial component of the diversity was more diverse than the fungal component (Figure 5A–C). Herein, *Rhizobiales* was the most abundant order present in decayed wood (22%) at T0. *Sphingomonadales*, *Sphingobacteriales* and *Rhodospirillales* orders represented respectively 13%, 12% and 11% of the bacterial diversity in decayed wood at T0 (Figure 5A). *Proteobacteria* and *Bacteroidetes* were thus the main phyla inhabiting this wood type (20% and 57%, respectively). Regarding the genus level, *Staphylococcus* were present in decayed wood at T0 with an inconsistent abundance but were much more represented in median and sound wood (11% in both wood types) (Figure 5B). *Burkholderia* genus was also more abundant in median and sound wood at T0 (8% and 6%) compared to decayed wood (1%). *Enterobacteriales*, namely *Enterobacter* spp. and *Pantoea* spp. were present in all wood types but in a low abundance at T0; the wood type in which *Enterobacteriales* were more present at T0 was median wood (10%) (Figure 5A,C). Conversely, *Enterobacteriales* represented most of the bacterial diversity at T3 (51% in median and 33% in sound wood). Focusing on the *Enterobacteriaceae* family at T3, *Enterobacter* spp. were the most abundant in median wood (44%), while *Pantoea* spp. were present in sound wood (26%) (Figure 5B). *Burkholderiales* order, represented by *Burkholderia* (*sensu lato*) and *Massilia* genera, grew significantly from T0 to T3, covering the 7% and the 13% in median and sound, respectively. *Rhodospirillales* presence did not change over time in both median and sound wood while *Pseudomonadales*, namely *Pseudomonas* spp., which were not relevant in T0, became representative at T3 in both median (3%) and sound (5%) wood (Figure 5C).

#### 3.2.2. Alpha-Diversity Analysis

**Figure 6:**
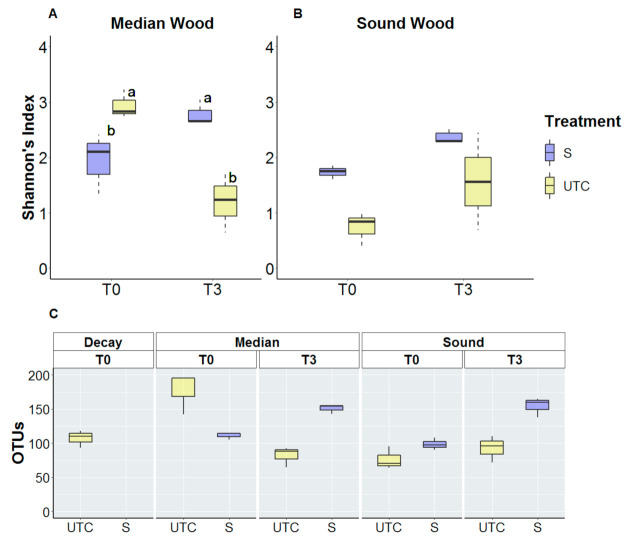
Fungal Alpha-diversity of trunk-surgery treated and untreated vines that showed LS symptoms in the previous year is measured by Shannon’s index (**A**,**B**) and by Observed Richness (**C**). Treatments are compared for both median (**A**) and sound (**B**) wood before trunk surgery (T0) and 3 months after (T3). Trunk surgery-treated vines (S) and untreated vines (UTC) are compared. Statistical analysis was performed on Shannon’s index values using a 2-way ANOVA test (α < 0.05) and Duncan post-hoc was carried out only for median wood where interaction between factors (Time and Treatment) was significant.

Fungal alpha-diversity of untreated vines (UTC) was higher in median wood than in sound wood at T0, while in T3 they tended to present similar values as shown in (Figure 6). While from T0 to T3, a natural significant decrease was observed in median wood, compared to T0, the fungal diversity increased slightly in sound wood. Focusing on trunk surgery-treated vines, the fungal diversity increased in median wood from T0 to T3 conversely to untreated control while as for non-treated vines, it increased in sound wood with a similar trend. Shannon’s index analysis highlights the inverse behavior in median wood where alpha-diversity of untreated vines decreased from 2.93 to 1.20 while values of trunk-surgery treated vines passed from 1.93 to 2.78 (Figure 6A,B). Moreover, the Observed Richness confirms that phenomena: detected OTUs in untreated vines decreased from 177 to 81, while increased from 111 to 150 in trunk surgery-treated vines in median wood (Figure 6C).

**Figure 7:**
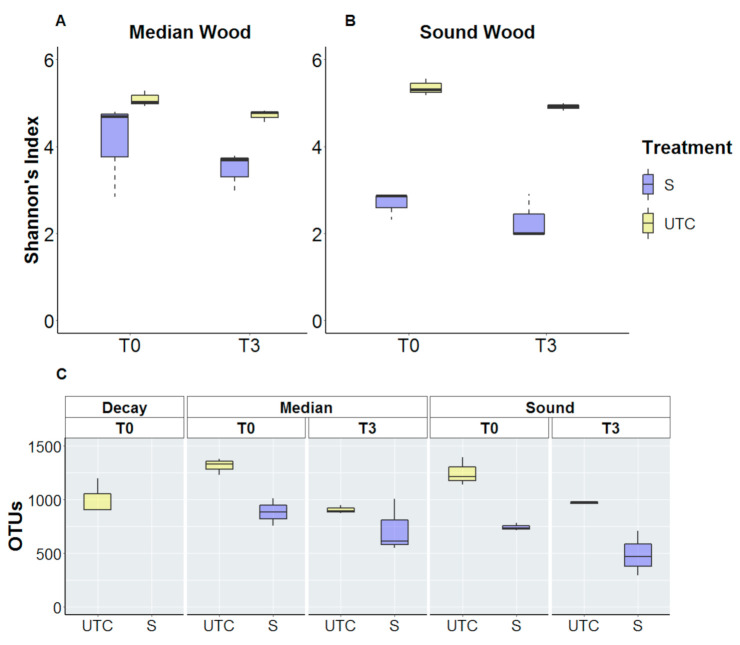
Bacterial alpha-diversity of trunk-surgery treated and untreated vines that showed LS symptoms in the previous year is measured by Shannon’s index (**A**,**B**) and by observed Richness (**C**). Treatments are compared for both median (**A**) and sound (**B**) wood before trunk surgery (T0) and 3 months after (T3). Statistical analysis performed on Shannon’s index values using a two-way ANOVA test (α < 0.05) did not highlight significant differences between T0 and T3 in both median and sound wood.

Overall, the alpha-diversity of bacterial microbiota decreases from T0 to T3 in both median and sound wood and for both trunk surgery-treated (S) and untreated vines (UTC). Shannon’s index values of untreated vines decreased from 5.09 to 4.71 in median wood, and from 5.36 to 4.91 in sound wood. Shannon’s index values of trunk surgery-treated vines decreased from 4.11 to 3.46 in median wood and from 2.68 to 2.29 in sound wood (Figure 7A,B). Compared to fungal diversity, bacteria values of detected OTUs were largely higher: at T0, 1314 and 1251 OTUs were identified for untreated vines in median and sound wood, respectively (Figure 7C). In general, starting from a comparable situation at T0 in median wood, bacterial biodiversity at T3 decreased more in treated vines than in untreated vines, while the genera abundance changed less between treated and untreated in sound wood over time. 

#### 3.2.3. Beta-Diversity

**Figure 8:**
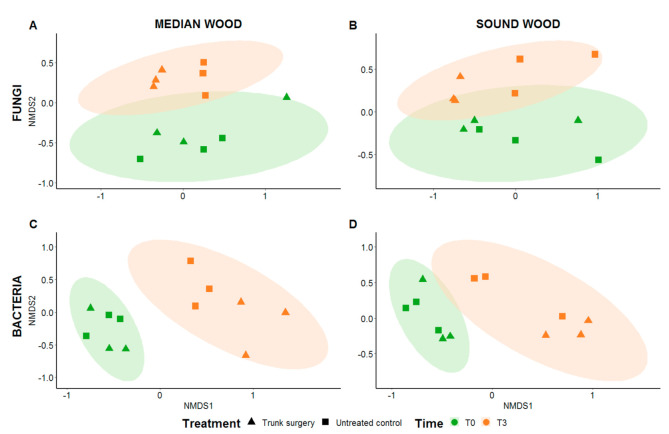
Non-metric multidimensional scaling (NMDS) analysis of fungal (**A**,**B**) and bacterial (**C**,**D**) microbiota. Microbiota of trunk surgery-treated vines and untreated control were analyzed over two timepoints (T0 and T3) considering 674 OTUs for fungi and 5676 OTUs for bacteria. Ellipses indicate 95% confidence intervals fitted into the spatial ordination. Microbial compositions of samples were significantly different where ellipses do not overlap. Significance was calculated performing ANOVA analysis (*p* < 0.05).

The analysis of beta-diversity highlighted the homogeneity of fungal and bacterial composition of all samples before trunk surgery treatment at T0 for both median and sound wood (Figure 8). However, after 3 months from trunk surgery (T3), the microbiota composition of the treated vines tended to differ from the microbiota of the untreated ones. Overall, fungal microbiota appeared to be more affected by trunk surgery compared to bacterial microbiota, which was represented mainly in sound wood. 

#### 3.2.4. Taxa Variation across Time

**Figure 9:**
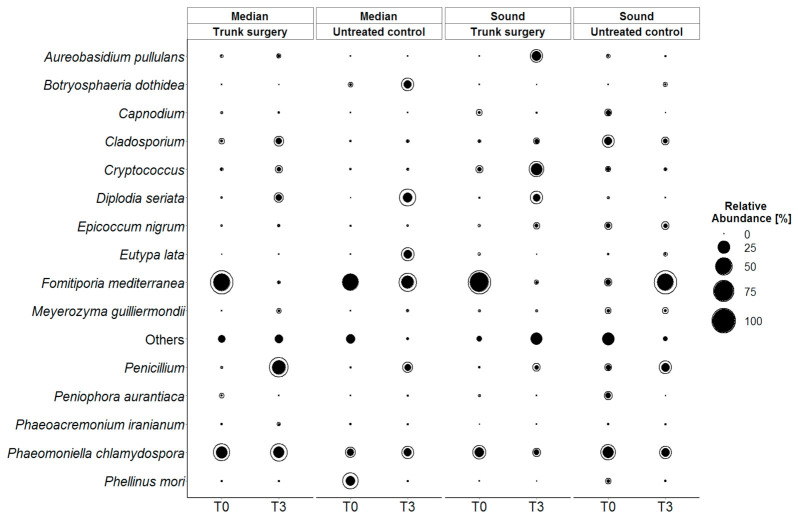
Qualitative changes in fungal diversity grouped by wood types (median and sound) a day before trunk surgery (T0) and 3 months after (T3); only the taxa with a relative abundance greater than 1% and the taxa which were much relevant for grapevine are showed. Solid black circles represent mean relative abundance of three biological repetition and external black line describes + standard deviation (SD): the greater the SD, the bigger is the space between the black line and the black solid circle.

To better understand the effect of trunk surgery on the microbiota, the relative abundance of the most representative taxa was analyzed over timepoints (T0 and T3). Eleven of the most abundant fungal taxa belonged to *Ascomycota*, while four belonged to *Basidiomycota*. As shown in Figure 9, grapevine pathogens were most abundant in sound wood. Focusing on pathogens involved in the diseases of the Esca complex, *P. chlamydospora* (Pch) was present in both median and sound wood in a similar manner and its presence appeared not to be affected by trunk surgery. Pch abundance did not change (from T0 to T3) comparing trunk surgery treated and untreated vines. Conversely *Fomitiporia mediterranea* (Fmed) was the species that showed a stronger variation, decreasing significantly from T0 to T3 in both treated median wood and treated sound wood. On the other hand, Fmed abundance increased in untreated sound wood and remained high in untreated median wood. The other phytopathogenic species, *Diplodia seriata* and the *Penicillium* genus were more abundant at T3 compared to T0 in both wood types shown to be affected by time more than by trunk surgery. *Eutypa lata* and *Botryosphaeria dothidea* appeared consistently only in median wood at T3. Cryptococcus sp. and *Aureobasidium pullulans* abundance increased significantly in treated vines compared to untreated control after treatment in both median and sound wood. 

**Figure 10:**
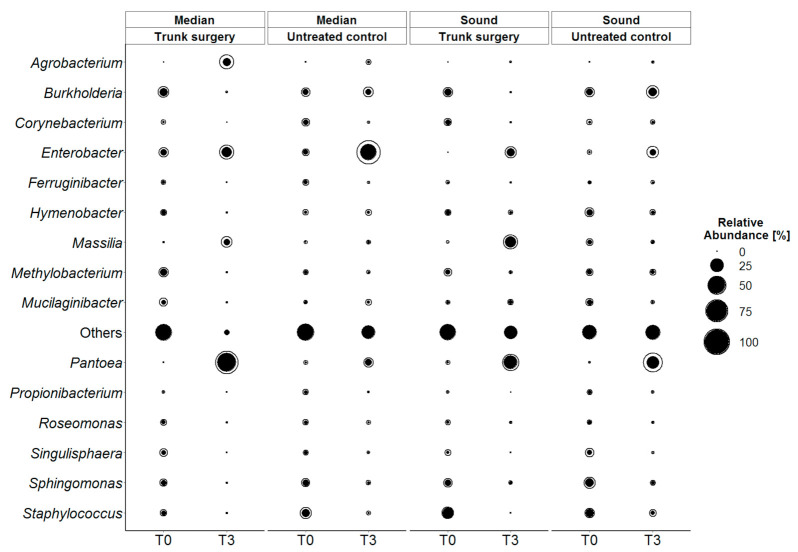
Qualitative changes in bacterial diversity grouped by wood types (median and sound) a day before trunk surgery (T0) and 3 months after (T3); only the genera with a relative abundance greater than 1% are showed. Solid black circles represent mean relative abundance of three biological repetition and external black line circles describe + standard deviation (SD): the greater the SD, the bigger is the space between the black line and the black solid circle.

The bacterial genus that has been affected most heavily by trunk surgery was *Burkholderia* (*sensu lato*), i.e., its abundance decreased in both median and sound trunk surgery-treated wood while its abundance did not vary in untreated control. The same was observed for the *Corynebacterium* genus but with lower values (Figure 10). Conversely to the former, *Massilia* sp. and *Pantoea* sp. increased considerably on median and sound treated vine woods. The abundance of members of the *Agrobacterium* genus increased with treatment in median wood but not in sound wood. Conversely, *Hymenobacter* genus decreased only in trunk surgery-treated median wood. As shown in Figure 10, other variations between bacterial microbiota can be noted for many genera over time but no consistent differences between treated and untreated emerged: *Propionibacterium*, *Roseomonas*, *Singulishaera*, *Sphingomonas* and *Staphylococcus* variation were more affected by time than by treatment.

## 4. Discussion

**Figure A1:**
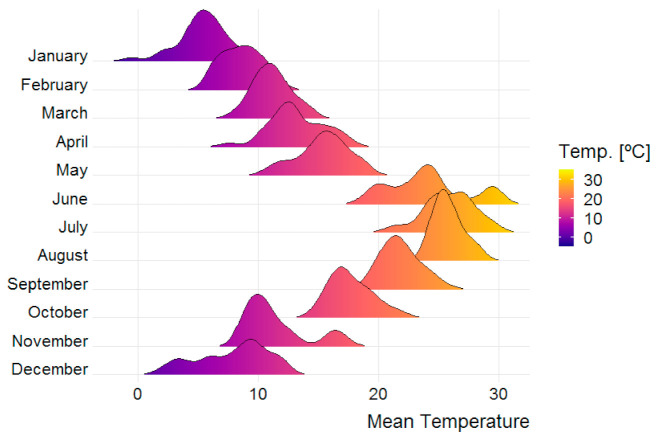
Mean Temperature (Celsius) recorded in the surveyed vineyard by month for 2019.

**Figure A2:**
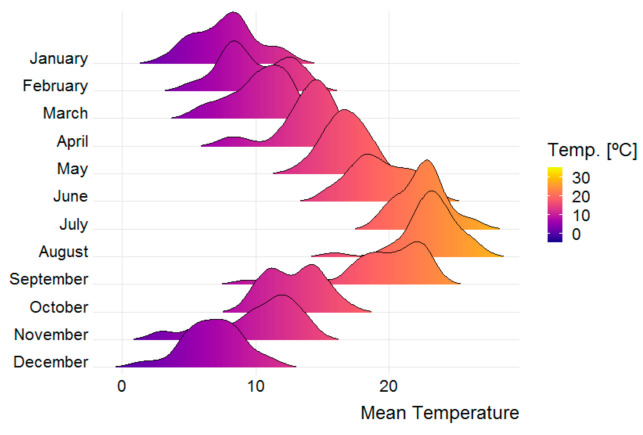
Mean Temperature (Celsius) recorded in the surveyed vineyard by month for 2020.

So far, only a few studies have been conducted on the efficacy of trunk surgery to reduce the expression of symptoms associated with Esca complex diseases, in particular with leaf stripe symptoms [51], and to our knowledge this is the first study that highlights the effect of the technique on the vine microbiota. It should be noted that the field tests were carried out over two years and characterized by different meteorological conditions (Appendix A, Figure A1 and Figure A2), and the consistency of the results obtained underlines their reproducibility. In order to better understand the role of decay removal on reducing foliar symptoms, three levels of treatment were tested. The trial showed that only the complete removal of decayed wood ensured the remission of LS symptoms in both years of trials, 2019 and 2020. Despite trunk surgery is currently being poorly described in the literature, the results obtained confirm the importance of the complete decay removal as suggested by other authors [36,37] and strengthen the hypothesis of a relevant role of decayed wood in foliar symptom expression [52]. Trunk surgery has been applied also on vines with wilted shoot symptoms and apoplexy: in those treatments, results showed, as to be expected, the inefficacy of the technique on reducing WS symptoms and moreover, highlighted a relevant increase of mortality on apoplectic vines probably due to the weakening of the vascular system. Comparing winter and summer treatment applications performed on vines showing LS symptoms in 2018, a higher number of dead vines was recorded in summer S-treatment, which may be due to the conjunction of the high rate of wood removal and the external climatic conditions (high temperatures and evaporation rate). This could be confirmed by the fact that only one Cut-treated vine and no HS-treated vines died in 2019 over the total of summer treated vines. Grapevine microbiota is influenced by various external factors, such as grape cultivars, farming practices, pedoclimatic conditions or geographical location [40,53,54]. Recently, several studies have been carried out on grapevine microbiota using different methodologies and the most relevant taxa of fungi and bacteria in grapevine wood are more defined [25,26,40,55]. The analysis on microbiota carried out in this experiment is related to a one-year study with the final aim of highlighting the fungal and bacterial taxa that were mainly affected by the trunk surgery technique. Obtained results allowed us to postulate hypothesis in order to carry out further investigations on the factors involved in the microbiota activity on symptoms appearance: a direct activity or a metabolite-driven activity. A first survey highlighted the microbiota composition of three analyzed wood types—sound, median and decayed wood. The most abundant bacterial taxa found in decayed wood at T0 belong to *Rhizobiales*, *Sphingomonadales* and *Sphingobacteriales*. The number of bacterial taxa with a relative abundance higher than 1% decreased over time and the majority of median and sound wood bacterial diversity were represented by few taxa: *Enterobacteriales*, *Burkholderiales*, *Pseudomonadales* and *Rhodospirillales* being the most abundant orders at T3. The most representative fungal taxa detected in decayed wood consisted mainly of pathogenic species, in which *F. mediterranea* and *P. chlamydospora* were the most abundant species found in decayed wood as previously shown by different authors [10,25,26,56]. Pmin, which is a common species often detected in Esca affected vines, was not found at all in the analyzed samples. Within the genus *Phaeoacremonium*, only *P. iranianum* was present in all the studied wood types with a very low relative abundance, a species that has been associated to Esca complex diseases only occasionally [57,58,59]. The analysis of the microbiota composition before treatment highlights that median wood fungal diversity was represented by a higher number of OTUs than sound wood fungal diversity due to a higher number of taxa in median wood. As a consequence of trunk surgery treatment, the fungal diversity raised in both median and sound wood of the treated vines, while it increased only in sound wood in the untreated control as shown by alpha-diversity analysis. Conversely, bacterial diversity decreased from T0 to T3 in a similar manner in both median and sound wood, without relevant differences between treated and untreated vines. Moreover, clusters observed at T3 in beta-diversity analysis showed how trunk surgery appears to select specific taxa suggesting the opportunity to investigate the role of excluded taxa in symptom development and at the same time on the increase in the population of bacterial species belonging to *Pantoea* spp. and the yeast-like fungus *Aureobasidium pullulans* in the vines showing symptom reduction. Among bacteria, *Burkholderia* (*sensu lato*) decrease in trunk surgery-treated wood (both median and sound wood). *Corynebacterium* decrease too while *Massilia* and *Pantoea* increased. *Burkholderia sensu lato* comprise several genera, such as *Trinikia, Paraburkholderia, Burkholderia, Caballeronia* and *Mycetohabitans* [60]. Some of these genera have been previously detected in grapevine. Some strains of *Burkholderia*, *Massilia* and *Pantoea* have been reported to be endophytic in grapevine plant tissues and associated with biocontrol action against phytopathogens [61,62]. Changes of other taxa abundance were detected suggesting that trunk surgery impacts ecologic niches of these microbial endophytes and that some of them are better adapted to survive in the specific wood tissue. Some other taxa are more affected by time and plant physiology with differences according to plant tissue [55] including trunk [63]. Obtained results highlight that trunk surgery allows for the enhancement of fungal diversity and the selection of specific taxa in median and sound wood. Analyzing the change of the most abundant species over time, Fmed is the fungal specie most affected by trunk surgery beside being the most recurrent species detected in decayed wood before trunk surgery application. Applying trunk surgery, the decayed tissue—which acts as a sort of fungal mycelium reservoir—is removed and Fmed presence decreases significantly in both the remaining median and sound wood showing a correlated behavior with symptom remission. This phenomenon led us to postulate that the Fmed activity in wood colonization had a relevant role in foliar symptom expression in “Esca proper” affected vines. Further investigations could be devoted to evaluate the possible role of light and air, as well as plant physiological reaction. Moreover, arsenite treatments reduced the expression of LS symptoms and it was found that arsenite concentrates in the decayed wood tissue where it showed a fungal inhibiting activity [64,65]. Considering this evidence, it is reasonable to accept that *Fomitiporia mediterranea* and its metabolism can play a main role in LS symptom expression. Furthermore some of the results obtained suggest that this basidiomycete might not require previous infection in order to extensively colonize the wood as recently highlighted by other authors [66]. On the other hand, the hypothesis that a single fungus causes the chloro-necrotic symptoms on leaves still cannot be accepted, despite it appears strictly related to Fmed presence in the rotten wood, as the same foliar symptoms are well known to be present in vines that have no wood rot or decay and so no basidiomycetes activity [67,68,69]. This suggests that there are some pathways in common among extremely different pathogens that cause a similar reaction, and therefore symptomatology, on the vine. While Esca in its original definition describes a wood rot, the association with leaf stripe symptoms has been better described as a separate disease, namely GLSD, that clearly appears to be due to various factors, and not to the action of a single pathogen. The idea of synergism between pathogens as a main trigger responsible for disease onset has been recently proposed by Bruez [70]. Recent literature reviews on wood degradation mechanism shows how some white rot, brown rot and soft rot agents share a non-enzymatic iron-dependent mechanism to intake lignocellulose biomass, either acting alone or in synergism with enzymes [71,72]. The involvement of those mechanisms in Esca related fungi has been proposed for Pch and Pmin [73], as well as for Fmed [74]. Furthermore, the evidence on the role of phytotoxic metabolites which could also contribute to the expression of symptoms [75,76] interacting with the aforementioned pathways, or alone, cannot be excluded. Thus, it is possible to hypothesize that the role played by those pathways could be very relevant in causing the foliar symptom expression, better described as a separate disease from white rot within Esca complex of diseases. Further research on the topic is needed to confirm this hypothesis and investigate the role of the metabolites involved in vine physiology.

## 5. Conclusions

Trunk surgery is a technique applied to reduce leaf stripe symptoms when they occur in vines, showing the whole range of wood symptoms and especially wood decay (the condition that can be described as Esca proper). This study shows the efficacy of the trunk surgery in LS symptom remission suggesting a link between the fungal wood colonization by basidiomycetes and foliar symptoms. This is highlighted by the fact that the higher was the degree of decayed wood removal, the lower was the expression of LS foliar symptom shown. This study strengthens the knowledge on vine microbiota and for the first time describes changes induced on it by trunk surgery technique as well as highlights the link between the decrease of the expression of LS symptoms and the abundance of *Fomitiporia mediterranea*.

## Data Availability

The data presented in this study are available within the article and the supplementary materials.

## Appendix A

**Table A1:** Microbiota of each different type of wood before trunk surgery. Listed species represent 96% of fungal diversity in decayed wood, 85.5% in median wood and 99% in sound wood. Only the species with a RA greater than 1% and the species which were much relevant for grapevine are shown. Wood type: ■ Decay wood, ▲Median wood, ● Sound wood.. Ecology in wood: (E) Endophyte; (P) Pathogen; (S) Saprotroph; (U) Unknown.

Species	Phylum	Family	Ecology in Wood	Wood Type	Relative Abundance (%)	References
*Acremonium* sp.	*Ascomycota*	*Incertae-sedis*	E, P	▲	0.2	[77,78,79,80,81,82,83]
*Alternaria* sp.	*Ascomycota*	*Pleosporaceae*	E, P	▲; ●	0.1; 0.1	[17,78,80,82,84,85,86,87,88]
*Aureobasidium pullulans*	*Ascomycota*	*Saccotheciaceae*	E, S	■; ▲	0.3; 0.9	[28,80,87,89,90,91,92,93,94]
*Botryosphaeria dothidea*	*Ascomycota*	*Botryosphaeriaceae*	E, P, S	■; ●	0.2; 1.4	[28,78,79,91,92,94,95,96,97,98,99,100,101,102,103,104,105,106,107,108,109]
*Botrytis cinerea*	*Ascomycota*	*Sclerotiniaceae*	E, P, S	▲	0.2	[28,80,87,88,91,92,96,110,111,112,113,114,115,116,117,118,119,120]
*Capnodium* sp.	*Ascomycota*	*Capnodiaceae*	P	■; ▲	0.3; 4.7	[121]
*Capronia* sp.	*Ascomycota*	*Herpotrichiellaceae*	S	■; ▲	5.2; 0.7	[122]
*Cladosporium* sp.	*Ascomycota*	*Cladosporiaceae*	E, P, S	■; ▲; ●	0.5; 9.1; 0.2	[28,88]
*Cladosporium sphaerospermum*	*Ascomycota*	*Cladosporiaceae*	P, S	▲	1.1	[88]
*Cryptococcus* sp.	*Basidiomycota*	*Cryptococcaceae*	E, S	■; ▲; ●	0.3; 2.8; 0.2	[28,92]
*Diplodia seriata*	*Ascomycota*	*Botryosphaeriaceae*	E, P, S	■; ▲	0.2; 0.3	[78,79,82,88,91,92,94,97,98,102,104,108,123,124,125,126,127,128,129,130,131]
*Epicoccum nigrum*	*Ascomycota*	*Pleosporaceae*	E, S	■; ▲; ●	0.1; 4.3; 0.1	[80,88,91,92,96]
*Eutypa lata*	*Ascomycota*	*Diatrypaceae*	P	■; ▲	10.8; 0.2	[17,79,103,114,121,130,132,133,134,135,136,137,138,139,140,141,142]
*Exophiala *sp.	*Ascomycota*	*Herpotrichiellaceae*	E	■; ▲	2.0; 0.7	[28]
*Fomitiporia mediterranea*	*Basidiomycota*	*Hymenochaetaceae*	P	■; ▲; ●	45.0; 5.9; 55.0	[6,79,82,130,143,144,145,146]
*Leptosphaeria* sp.	*Ascomycota*	*Leptosphaeriacea*	E; S	▲	0.1	[80,88]
*Meyerozyma guilliermondii*	*Ascomycota*	*Debaryomycetaceae*	E	■; ▲	0.1; 2.3	[147,148]
*Penicillium* sp.	*Ascomycota*	*Aspergillaceae*	E; P; S	■; ▲	0.9;5.5	[28,80,82,88,92,123]
*Peniophora aurantiaca*	*Basidiomycota*	*Peniophoraceae*	S	■; ▲	11.7; 5.7	[149,150]
*Phaeoacremonium iranianum*	*Ascomycota*	*Togniniaceae*	P	■; ▲; ●	1.0; 0.2; 0.1	[57,151,152,153,154,155,156]
*Phaeomoniella chlamydospora*	*Ascomycota*	*Phaeomoniellaceae*	P	■; ▲; ●	17.2; 23.3; 9.4	[3,17,78,79,88,107,130,154,157,158,159,160,161,162]
*Phellinus mori*	*Basidiomycota*	*Hymenochaetaceae*	U	■; ▲; ●	0.1; 2.3; 17.6	
*Pleospora herbarum*	*Ascomycota*	*Pleosporaceae*	E	▲	2.2	[92]
